# The Impact of Chat Generative Pre-trained Transformer (ChatGPT) on Oncology: Application, Expectations, and Future Prospects

**DOI:** 10.7759/cureus.48670

**Published:** 2023-11-11

**Authors:** Yanxing Li, Wentao Gao, Zhenhua Luan, Zhi Zhou, Jianjun Li

**Affiliations:** 1 Department of Clinical Oncology, Xi'an Jiaotong University Health Science Center, Xi'an, CHN; 2 Department of Cardiology, Jincheng People's Hospital, Changzhi Medical College, Jincheng, CHN

**Keywords:** diagnosis, cancer, oncology, chatgpt, artificial intelligence

## Abstract

The integration of Chat Generative Pre-trained Transformer (ChatGPT) into the field of oncology has recently garnered significant attention, with potential implications for enhancing both research and clinical practice. This paper presents a comprehensive review of the evolving landscape of ChatGPT applications in oncology, emphasizing its contributions to diagnosis, treatment, research, and patient education. We examine its role in assisting medical professionals, researchers, and patients in understanding complex cancer-related data and making informed decisions. Ethical considerations and potential challenges associated with the implementation of ChatGPT in oncology are also discussed. This article highlights the promising role of ChatGPT as a valuable tool in the oncology domain and outlines future directions for research and application.

## Introduction and background

Chat Generative Pre-trained Transformer, or ChatGPT, is a pioneering achievement in artificial intelligence (AI). This versatile AI model is designed to understand and generate human-like text, fundamentally transforming how we interact with language. Its applications span various domains, making it an invaluable tool for a wide range of tasks [1].

In the context of health sciences, where the volume of medical knowledge is growing exponentially, ChatGPT plays a transformative role [2,3]. It simplifies the process of retrieving precise and up-to-date information, revolutionizing how professionals access the latest research and insights [4,5]. ChatGPT's influence extends beyond the realm of health sciences, impacting specialized medical fields like radiology, pathology, and genomics, by simplifying complex medical terminology and facilitating effective communication with patients [6-9].

**Figure 1:**
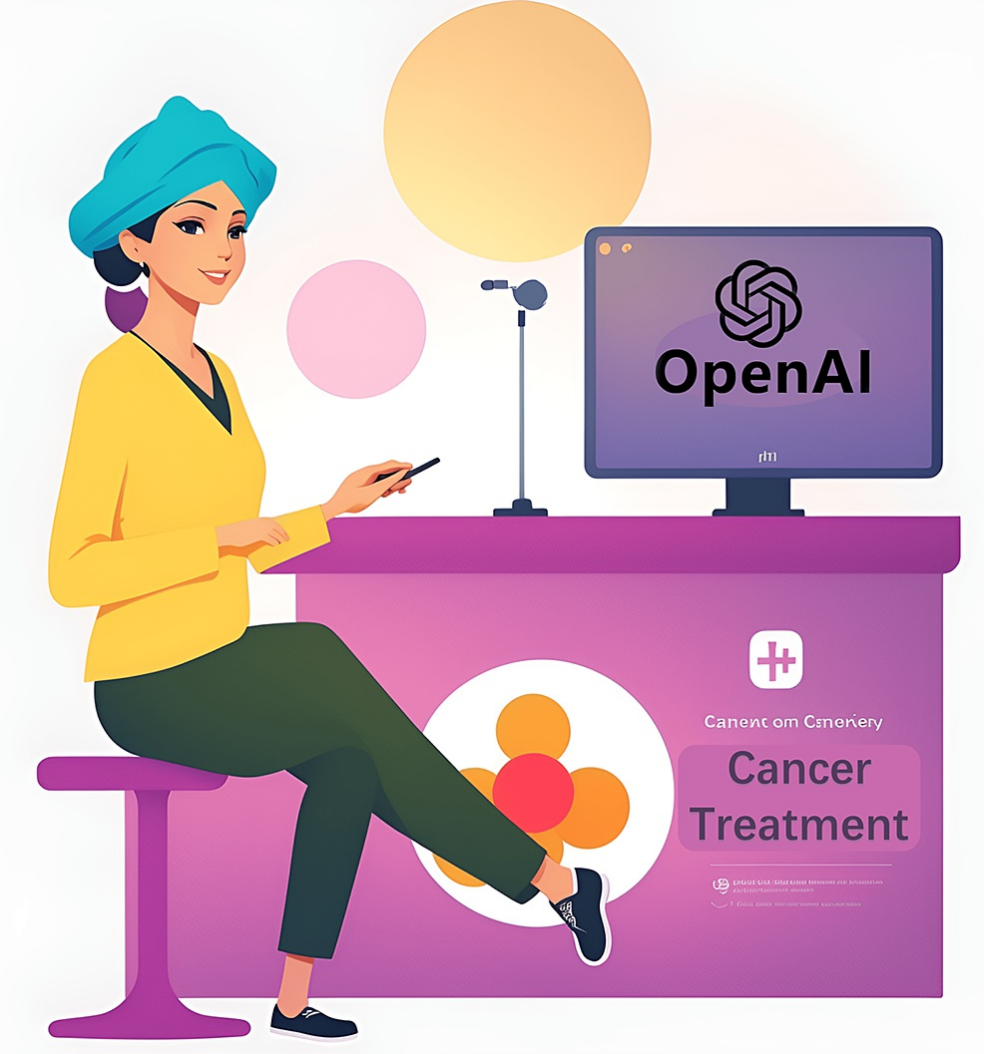
ChatGPT impacts on cancer treatment.

Turning our attention to oncology, ChatGPT emerges as a game-changer. Cancer, a leading global cause of mortality, presents multifaceted challenges in diagnosis, treatment, and research [10]. Recent advancements in AI and natural language processing have opened new frontiers in the oncology landscape, with ChatGPT at the forefront [11-13]. Integrating ChatGPT into oncology offers a promising opportunity to enhance medical research, clinical decision-making, and patient education [14-16]. ChatGPT's unique ability to generate human-like text and comprehend complex medical literature equips medical professionals, researchers, and patients to navigate the intricate landscape of cancer-related information. This paper comprehensively explores the evolving role of ChatGPT in oncology, shedding light on its multifaceted applications, implications, and prospects (Figure 1).

## Review

ChatGPT in cancer diagnosis

Cancer diagnosis is a complex and critical task that relies on various medical data sources, including medical imaging, pathology reports, and genomic information. The integration of ChatGPT into oncology practice has shown considerable promise in supporting medical professionals during cancer case analysis and diagnosis [17,18].

ChatGPT, with its natural language processing capabilities, has the potential to assist oncologists and pathologists in interpreting medical records, patient histories, and clinical notes [15,16]. It can effectively summarize patient information and provide relevant insights into potential diagnoses. For example, when presented with a patient's medical history, ChatGPT can assist in identifying relevant risk factors, genetic predispositions, and potential diagnostic considerations, empowering oncologists to make more informed decisions [8,19].

A crucial aspect of cancer diagnosis is the interpretation of medical imaging data, such as X-rays, MRIs, and CT scans. ChatGPT plays a significant role in aiding radiologists and oncologists in this process. When provided with medical images, ChatGPT can assist in generating descriptive reports that highlight potential abnormalities, tumor characteristics, and areas of concern. This can improve the accuracy and speed of diagnosing cancerous conditions [6,7]. The application of ChatGPT in medical imaging interpretation extends to areas like mammography, where early detection of breast cancer is paramount. By analyzing mammograms, ChatGPT can identify suspicious regions and offer insights to radiologists, potentially enhancing early diagnosis rates [12,20,21].

Pathology reports, which offer detailed information about tissue samples and biopsies, are indispensable in cancer diagnosis and classification. ChatGPT can support pathologists by summarizing and interpreting these reports. When presented with a pathology report, ChatGPT can identify key findings, highlight relevant details, and suggest potential cancer types or stages based on the provided information. This streamlines the diagnostic process, minimizes errors, and enhances consistency in pathology assessments [22].

For example, when a pathologist reviews a tissue biopsy report, ChatGPT can assist in summarizing the report's findings. It can highlight key details, such as the presence of abnormal cells, tissue characteristics, and potential cancer markers. This summary can aid pathologists in their assessments, ensuring that critical information is not overlooked and that the diagnostic process is efficient and thorough.

Genomic data, including genetic mutations and alterations, play a vital role in understanding cancer development and personalizing treatment plans. ChatGPT can be instrumental in assisting oncologists and genetic counselors in interpreting genomic data. By analyzing genetic reports and sequencing data, ChatGPT can identify known genetic markers and provide information about their clinical significance. This facilitates the selection of targeted therapies and helps patients and healthcare providers make informed decisions about treatment options [8,19].

As an illustration, suppose a patient's genomic data reveals specific genetic mutations associated with a particular cancer type. ChatGPT can analyze this information and provide insights into the clinical significance of these mutations. It can suggest targeted therapies and potential treatment options based on the patient's unique genetic makeup. This empowers oncologists and genetic counselors to make personalized treatment recommendations that align with the patient's genetic profile.

ChatGPT in cancer treatment

Predicting the prognosis of cancer patients and offering personalized treatment recommendations are critical elements of modern oncology. ChatGPT holds substantial potential in enhancing these aspects, ultimately improving patient outcomes and advancing cancer research.

ChatGPT can be a valuable tool for predicting the prognosis of cancer patients by analyzing their medical records, which may include histopathology reports, imaging data, and genetic profiles. By processing this data, ChatGPT can generate forecasts regarding disease progression, potential risks, and patient survival probabilities. This information can assist oncologists in developing personalized treatment plans and facilitating discussions about prognosis with patients and their families [18].

The efficacy of cancer treatments varies from one patient to another due to differences in tumor characteristics, genetics, and individual responses. ChatGPT can aid in providing personalized treatment recommendations by analyzing a patient's medical history, test results, and known treatment responses. It can generate suggestions regarding the most appropriate treatment options, potential side effects, and expected outcomes. These recommendations can empower healthcare providers and patients to make decisions that align with the patient's unique condition [18,23].

For instance, consider a situation where a patient is exploring treatment options for their cancer diagnosis. ChatGPT can analyze the patient's medical history, including details about the type and stage of cancer, previous treatment responses, and any genetic information. Based on this data, ChatGPT can generate personalized treatment recommendations, taking into account the patient's specific condition and potential responses to different therapies. This personalized guidance can aid healthcare providers and patients in making treatment decisions that are tailored to the individual's needs.

However, as ChatGPT becomes integrated into cancer prognosis and treatment recommendations, ethical considerations and regulatory compliance assume paramount importance. Ensuring that ChatGPT's recommendations are rooted in validated, evidence-based sources is essential. Additionally, safeguarding patient data security and privacy is of the utmost importance when employing AI in clinical decision-making. Adherence to regulatory guidelines and the active involvement of medical professionals in decision-making processes are vital aspects of the ethical implementation of ChatGPT in oncology [1,24,25].

For ethical considerations, imagine a scenario where ChatGPT is being used to provide treatment recommendations to a patient. It is crucial that the recommendations are based on the latest evidence and validated sources to ensure the highest quality of care. Furthermore, patient data privacy and security must be rigorously maintained to protect sensitive medical information. Regulatory compliance and the active participation of medical professionals in decision-making are fundamental to the responsible and ethical use of ChatGPT in oncology.

ChatGPT in cancer patient education

Empowering cancer patients with a clear understanding of their diagnosis and treatment options is pivotal for informed decision-making and enhancing the overall quality of care. ChatGPT presents the potential to serve as a valuable tool for providing accessible and patient-friendly cancer information and support.

ChatGPT can play a crucial role in helping patients comprehend complex cancer diagnoses and the multitude of available treatment options. When presented with a patient's medical history, diagnosis, or treatment plan, ChatGPT can generate plain language explanations and descriptions, avoiding jargon and complex medical terminology that might pose challenges for patients. This feature promotes clarity and alleviates anxiety for individuals facing a cancer diagnosis [14,26,27].

Cancer is a profoundly heterogeneous disease, and treatment decisions often necessitate personalization based on an individual's medical history and preferences. ChatGPT can personalize the delivery of cancer information by tailoring responses to specific patient profiles. By understanding a patient's medical history and preferences, ChatGPT can provide targeted information on available treatment options, potential side effects, and expected outcomes [23].

Cancer treatment decisions are often intricate and emotionally charged. ChatGPT can assist patients in making informed choices by offering information regarding the advantages and risks associated with different treatments. Moreover, it can address patients' inquiries about treatment processes, potential side effects, and lifestyle adjustments during cancer treatment. This support empowers patients to actively engage in their care decisions [9].

However, the utilization of ChatGPT in patient education does raise ethical concerns pertaining to privacy and data security. Ensuring the protection of patient data and adherence to relevant privacy regulations is of utmost importance. Patients must also be informed about the use of AI tools in their care and have the option to opt in or out of such interactions [1,25].

ChatGPT in cancer research

In the field of medicine, staying updated with the constant influx of new studies and publications presents a challenge for researchers, making comprehensive literature reviews a demanding task. ChatGPT emerges as a promising solution, offering an efficient means to review extensive medical research literature, transcending its impact beyond oncology.

ChatGPT's natural language understanding and generation capabilities have the potential to enhance the efficiency of literature search and retrieval across various medical specialties. By allowing researchers to input queries in natural language, it ensures comprehensive and relevant results, irrespective of the specific medical domain. Researchers can save valuable time and effort as ChatGPT comprehends context and provides nuanced search results, eliminating the need for complex and structured search queries [4,28,29].

Furthermore, ChatGPT's capabilities extend beyond merely accessing existing medical literature. It can suggest new research directions and generate hypotheses, fostering a conversational engagement with researchers. Drawing upon extensive medical knowledge, ChatGPT can propose novel research avenues, identify gaps in current knowledge, and even contribute to the formulation of research hypotheses. This opens up exciting opportunities in medical research, enabling scientists to explore uncharted territories [30,31].

Once the relevant literature is retrieved, ChatGPT aids researchers in extracting key information and summarizing research findings, not limited to oncology but extending to fields like cardiology, neurology, or infectious diseases. This includes the generation of concise research article summaries, highlighting methodologies, results, and conclusions. Moreover, ChatGPT can identify trends, commonalities, and disparities in the literature, simplifying the synthesis of the current state of knowledge in any medical discipline [5,28].

ChatGPT's capabilities are invaluable in assisting with meta-analysis across various medical research areas. Meta-analyses often require the extraction and aggregation of data from numerous studies, spanning diverse domains from oncology to pediatrics. ChatGPT streamlines the process, automating data extraction and summarization, reducing the potential for errors, and expediting meta-analysis across a spectrum of medical domains [32,33].

Beyond literature review, ChatGPT plays a pivotal role in guiding researchers in identifying potential drug targets and designing clinical trials across a wide array of medical specialties. It can analyze extensive datasets, including genomic information, clinical trial data, and medical literature, providing insights and recommendations for developing novel therapies and research designs. Whether researchers are focused on cancer or cardiovascular diseases, ChatGPT's data processing capabilities and cross-specialty applicability offer valuable support [34,35].

While ChatGPT presents significant potential in aiding researchers with literature review and meta-analysis, acknowledging its limitations and challenges is vital. These include the need for rigorous fact-checking, as ChatGPT's responses rely on the data it was trained on, which may not always be up-to-date or error-free. Additionally, ensuring that the automated process aligns with research quality standards and ethical guidelines is essential, irrespective of the medical domain under consideration [36].

Challenges and limitations of ChatGPT in real-world clinical environments

While ChatGPT presents remarkable potential in the field of oncology, its integration into real-world clinical settings is not without challenges and limitations.

Data Security and Privacy Concerns

One of the foremost challenges pertains to safeguarding patient data and privacy. The use of ChatGPT involves the processing of sensitive medical information, which raises concerns about data security. Ensuring the protection of patient data and compliance with relevant privacy regulations is paramount. Stricter measures and encryption protocols may be necessary to prevent data breaches or unauthorized access [1,25].

Ethical Considerations

The ethical implications of AI-driven decision support in clinical practice cannot be understated. ChatGPT's recommendations and insights need to align with the highest standards of medical ethics. Ensuring that its responses are rooted in validated, evidence-based sources is crucial. A careful review of ChatGPT's suggestions by medical professionals is essential to maintain ethical standards and avoid potential bias or misinformation [1,24].

Integration With Clinical Workflow

Real-world clinical environments are complex and often require seamless integration of AI tools into existing workflows. The successful adoption of ChatGPT in oncology clinics necessitates that it works harmoniously with electronic health records (EHRs) and other clinical systems. Achieving this integration without disrupting clinical operations is a challenge that requires careful planning and execution.

Rigorous Fact-Checking

ChatGPT's responses are based on the data it was trained on, and that data may not always be up-to-date or error-free. This limitation underscores the importance of rigorous fact-checking when using ChatGPT in clinical decision-making. Medical professionals must verify the information provided by ChatGPT to ensure its accuracy and relevance to each patient's unique case.

User Training and Familiarity

Healthcare professionals and support staff may require training to effectively use ChatGPT within their clinical workflows. Ensuring that healthcare providers are familiar with ChatGPT's capabilities, limitations, and ethical guidelines is essential for successful implementation.

Maintaining Patient Autonomy

While ChatGPT empowers patients with information, it is important to balance this with preserving patient autonomy in decision-making. Patients must not feel pressured or influenced by AI recommendations and should be encouraged to make choices aligned with their values and preferences.

Limited Understanding of Context

ChatGPT, while highly advanced, may still struggle to fully comprehend the intricate and nuanced context of each patient's case. This can result in responses that, while factually correct, may not align with the patient's unique situation. Human clinical judgment remains crucial in interpreting ChatGPT's recommendations.

Future directions of ChatGPT in oncology

The ongoing advancements in the field of AI and natural language processing offer promising opportunities for further harnessing ChatGPT's potential within the realm of oncology. This section delves into the potential future directions for ChatGPT's broader application in cancer care, including the integration of multimodal data and the augmentation of its self-learning capabilities.

Integration of Multimodal Data

One promising avenue for ChatGPT's application in oncology is the integration of multimodal data. This involves merging text-based information with diverse data types, such as medical images, genomics, and electronic health records. Through processing and synthesizing this wealth of data, ChatGPT can provide a more comprehensive understanding of a patient's condition. By combining medical images with clinical records, it can facilitate more precise and comprehensive cancer diagnoses. For example, when a patient's imaging data and clinical history are combined, ChatGPT can provide a more accurate assessment of the disease. This capability is particularly crucial in the context of personalized medicine. Furthermore, integrating genomic data with clinical profiles can lead to highly personalized treatment recommendations, as ChatGPT can analyze genetic information and suggest targeted therapies tailored to an individual's specific genetic makeup.

Enhanced Self-Learning Capabilities

The self-learning capabilities of ChatGPT can be further enhanced to provide more up-to-date and context-aware recommendations. ChatGPT can be designed to continually learn from real-world clinical data. By regularly updating its knowledge base with the latest medical literature, clinical trials, and patient outcomes, ChatGPT can offer insights based on the most recent evidence. Moreover, ChatGPT can be refined to better understand the clinical context and patient history during interactions, allowing it to provide more context-aware recommendations and explanations. This enhanced contextual understanding contributes to its clinical utility and supports healthcare professionals in making informed decisions.

Real-Time Collaboration With Healthcare Professionals

In the foreseeable future, ChatGPT could evolve into a sophisticated clinical decision support system that collaborates in real-time with healthcare professionals. This entails the seamless integration of ChatGPT into electronic health records and hospital systems. During patient visits, ChatGPT could assist healthcare providers by offering real-time guidance on diagnosis and treatment decisions based on the latest research and patient data. This functionality not only improves the quality of care but also saves valuable time for healthcare professionals. Furthermore, ChatGPT can automate the generation of clinical notes and reports, further streamlining administrative tasks and allowing healthcare professionals to focus more on patient care.

Enhanced Patient Engagement and Empowerment

The evolution of ChatGPT in oncology also encompasses enhancing its role in patient engagement and empowerment. User-friendly interfaces can be developed, allowing patients to directly interact with ChatGPT, obtain personalized health information, and monitor their progress. This dynamic interaction enables patients to actively participate in their care decisions. ChatGPT can serve as a resource for providing educational tools and materials to improve patients' health literacy and understanding of their medical conditions. This patient-centric approach not only enhances patient satisfaction but also contributes to better health outcomes and improved overall quality of care.

These future directions exemplify the potential for ChatGPT to continue revolutionizing the field of oncology by leveraging AI capabilities for more personalized and efficient healthcare. They offer a glimpse of how ChatGPT can evolve into a comprehensive tool that integrates seamlessly into the healthcare ecosystem, benefiting both patients and healthcare professionals.

## Conclusions

The integration of ChatGPT in oncology holds great promise, streamlining cancer research, diagnostics, and patient support. ChatGPT simplifies literature review and information retrieval for researchers, while aiding healthcare professionals in diagnosing and offering personalized treatment recommendations. Furthermore, its potential in drug development, patient education, and multimodal data integration signifies a transformative role in the field. However, ethical considerations and collaboration between stakeholders are imperative as ChatGPT shapes the future of oncology, ultimately enhancing the quality of cancer care and research.
